# Anti-cancer targets and molecular mechanisms of formononetin in treating osteosarcoma based on network pharmacology

**DOI:** 10.18632/aging.205139

**Published:** 2023-10-20

**Authors:** Lizhi Chen, Yue Zhou, Zheng Weng, Shuang Liu, Ting Li, Yanfang Wang, Yang Yang, Hongmei Liu, Wenhua Huang

**Affiliations:** 1 Department of Science and Education, Guangdong Second Provincial General Hospital, Guangzhou, Guangdong, China; 2 Guangdong Engineering Research Center for Translation of Medical 3D Printing Application, Guangdong Provincial Key Laboratory of Medical Biomechanics, National Key Discipline of Human Anatomy, School of Basic Medical Sciences, Southern Medical University, Guangzhou, Guangdong, China; 3 The Affiliated Guangdong Second Provincial General Hospital of Jinan University, Guangzhou, China; 4 Department of Hematology, Guangdong Second Provincial General Hospital, Guangzhou, Guangdong, China; 5 Department of Ultrasound, Institute of Ultrasound in Musculoskeletal Sports Medicine, Guangdong Second Provincial, General Hospital, Guangzhou, Guangdong, China; 6 Second School of Clinical Medicine, Southern Medical University, Guangzhou, Guangdong, China

**Keywords:** formononetin, osteosarcoma, network pharmacology

## Abstract

Osteosarcoma (OS) is a multifactorial bone malignancy that accounts for most cancers in children and adolescents. Formononetin has been proven to exhibit various pharmacological effects including anti-tumor, anti-obesity, anti-inflammation, and neuroprotective effects. Few studies have examined the pharmacological activities of formononetin in OS treatment, but the mechanism has not yet been completely elucidated. Network pharmacology is a new method based on the theory of system biology for analyzing the network of biological systems and selecting specific signal nodes for multi-target drug molecular design. Here, we used network pharmacology to explore the possible mechanism of formononetin in OS treatment. Human OS cell line MG63 was processed with four concentrations (0, 2, 5, 8 μg/mL) of formononetin. Subsequently, an MTT assay was performed to test cell proliferation and a scratch test was used to evaluate the migration ability of cancer cells. Caspase-3, p53, p21, and bcl-2 expression levels incubated with different concentrations of formononetin in MG63 cells were determined using Western blotting. After treated with formononetin for 48 h, MG63 cells exhibited marked apoptosis. The results revealed that certain concentrations of formononetin significantly exerted inhibitory effects on MG63 cell proliferation. Furthermore, formononetin decreased the bcl-2 level in MG63 cells but increased caspase-3, p21, and p53 levels in a concentration-dependent manner. Additionally, formononetin suppressed the expression of SATB2. Therefore, formononetin could dose-dependently inhibit MG63 cell proliferation and induce apparent cell apoptosis, providing a candidate treatment for OS, whereas SATB2 could be a potential prognostic biomarker for screening OS and therapeutic target of formononetin.

## INTRODUCTION

Osteosarcoma (OS) is a typical bone tumor with high mortality among children and adolescents [1, 2]. Its etiology involves malignant metastasis of mesenchymal stem cells (MCSs). In the last several decades, the overall survival rate of high-grade pediatric osteosarcoma has been greatly improved but clinically effective approaches for prevention and treatment of distant metastasis are limited [3]. To date, some efforts have been made to suppress OS, but only a few therapeutic strategies have notable recuperative and curative efficacy [4]. In clinical practice, chemotherapy, radiotherapy and surgery are commonly prescribed treatments for OS but these strategies often have adverse side effects [5]. Consequently, it is crucial to pursue safer and more effective medications for treating OS.

Formononetin is a bioactive isoflavone mainly isolated from Chinese medicinal plants, including Astragalus and licorice [6, 7]. Studies have also demonstrated that formononetin, a potent isoflavone phytoestrogen, has many promising pharmacological effects. By binding with estrogen receptor (ER), formononetin regulates gene expression and exerts various estrogen-dependent activities, such as anti-tumor, wound healing, antioxidant and anti-inflammatory effects [8–11]. Remarkably, formononetin effectively inhibits human cancer cell proliferation and accelerates the apoptosis of tumor cells in cervical tumors, colon carcinoma, gastric carcinoma, and breast cancers [12–15]. Moreover, it is searched that isoflavone phytoestrogens induce apoptosis in osteosarcoma cell lines via ERβ-mediated pathways, demonstrating that phytoestrogens have an anti-tumor effect on OS [16].

Network pharmacology is a new concept first put forward by British pharmacologist Andrew L. Hopkins [17]. It integrates multi-disciplinary knowledge including theories of systems biology, network informatics, genomics, and proteomics [18, 19]. It is helpful to analyze complex chemical components as well as their mechanism of action, to clarify the complex biological network relationship of drug component-disease-target network, and to verify the efficacy, side effects, and mechanism of action of drugs through experiments [20, 21]. Consistent with the multi-component, multi-channel, and multi-target characteristics of traditional Chinese medicine (TCM), network pharmacology provides a promising and efficient tool for TCM research. Recently, it has been extensively used to investigate relationships between TCM and diseases [22, 23]. Network pharmacology has also been applied in OS research to elucidate pharmacological mechanisms and treatment targets for OS [24, 25]. A network pharmacology-based research showed that formononetin might down-regulate ESR1, TP53, and ERBB2 positive expressions and reduce tumor weights in tumor-bearing nude mice hypodermically injected with OS cells [11].

**Figure 1:**
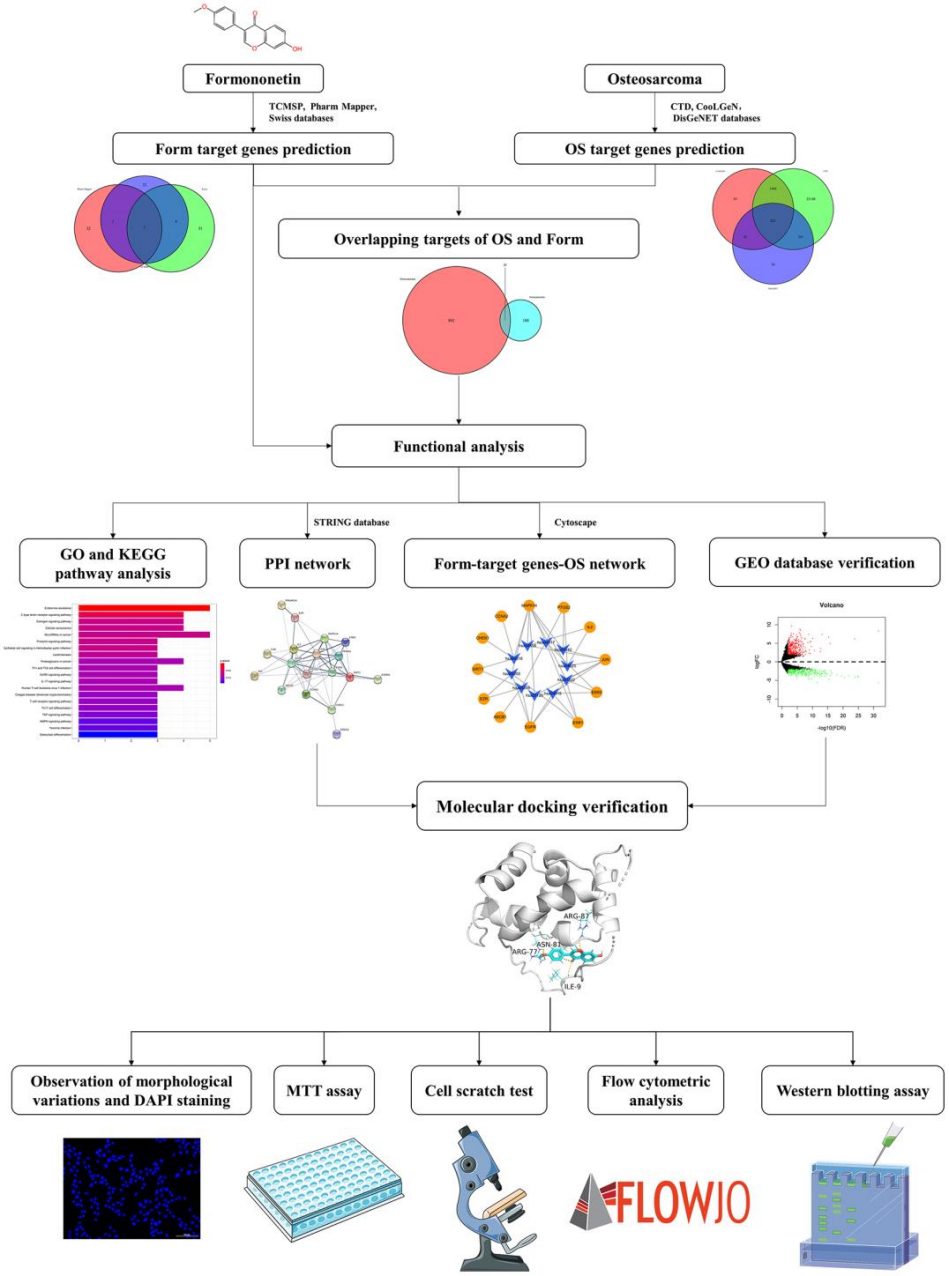
A workflow of network pharmacology analysis.

Therefore, we hypothesized that formononetin has some inhibitory effect on osteosarcoma and could serve as an effective anti-tumor therapeutic approach. Human osteosarcoma MG63 cells are a well-established osteoblastic cell line [26]. In this study, we used network pharmacology to study the anti-tumor activity of formononetin and determine its possible molecular mechanism in osteosarcoma treatment to provide a basis for further pharmacological action research and clinical application (Figure 1).

## MATERIALS AND METHODS

### Study design

We used network pharmacology to study the anti-tumor activity of formononetin and determine its possible molecular mechanism in osteosarcoma treatment to provide a basis for further pharmacological action research and clinical application.

### Screening of formononetin targets

Target proteins that interact with bioactive molecules of formononetin were obtained from TCMSP, DrugBanK (https://go.drugbank.com/), Pharm Mapper (https://pubchem.ncbi.nlm.nih.gov/), and Swiss databases (http://www.swisstargetprediction.ch/). We used the full name of the target protein to obtain the corresponding gene abbreviation via UniProt ID in the UniProt database. Intersection targets screened from the three databases were considered potential target protein targets.

### Functional analysis of target proteins

Gene ontology (GO) is a common method for annotation of genes and their expression products. GO functional analysis by DAVID database and pathway enrichment analysis based on clusterProfiler were performed. *P* < 0.05 was considered the critical value of significant gene enrichment. R software “clusterProfiler” (version 3.8) was used to make bubble charts for visualization. Kyoto encyclopedia of genes and genomes (KEGG) was searched to analyze the signal pathway of drug targets. The STRING database was used to build protein-protein interaction (PPI) network (http://www.string-db.org/) and Cytoscape software (version 3.7.2) was used to draw the drug targets-proteins network diagram. Network Analyzer was utilized to analyze three parameters of the network including degree, betweenness, and closeness.

### Screening of OS target gene

CTD (https://ctdbase.org/), CooLGeN, and DisGeNET (https://www.disgenet.org/) databases were used to establish a database of OS-related targets. The intersection targets of the three databases were considered promising OS target genes.

### Screening and functional analysis of common targets of OS and formononetin

Commonly predicted targets were searched from the combination of formononetin’s targets from TCMSP, Pharm Mapper, and Swiss databases and the intersection of OS’s targets from CTD, CooLGeN, and DisGeNET databases. GO functional analysis, KEGG pathway analysis, and PPI network analysis were used to analyze common targets.

### Construction of target-pathway pharmacology network of formononetin and OS

The OS protein interaction network was constructed by connecting OS-related proteins and other interacting proteins. Compound target OS target protein interaction network was constructed using the intersection compound of PPI target and OS PPI target. Cytoscape software (version 3.7.2) was used to draw a pharmacology network based on the relationship among ingredients, target proteins, and pathways.

### Validation of target protein using gene expression omnibus dataset

The OS mRNA chip dataset numbered GSE126209 was searched in the Gene Expression Omnibus (GEO) database of the NCBI, including six samples of OS patients and five samples of the healthy control group. The original GSE126209 dataset was downloaded and read with “Affy” R package (version 3.8). Subsequently, standardization preprocessing was performed with the RMA method, including background correction, standardization, and normalization. Probes were annotated with the platform annotation file to remove probes not matching the gene. For different probes mapped to the same gene, their mean value was taken as the final expression value of this gene. Samples were divided into two groups: the OS group and the healthy control group. The *p* value for expression difference and expression fold change value were calculated using limma package. *P* value < 0.05 and | log_2_FC | > 2 (four-fold relationship) were selected as the threshold for screening significantly differentially expressed mRNA. The volcano map and heat map were drawn by combining the differential gene expression matrix file. The protein target was the intersection of formononetin prediction target, multi-data osteosarcoma prediction target, and GEO differential gene.

### Molecular docking verification of core compounds and core target genes

AutoDock Vina was used to docking core targets downloaded from the PBD database and the chemical structure of formononetin downloaded from the PubChem database.

### Materials

Formononetin (purity ≥ 99.0%) and MTT and DAPI kits were purchased from Sigma (USA). Main antibodies Anti-p21 and anti-p53 were bought from Santa Cruz Biotechnology (USA). Anti-mouse and anti-rabbit-horseradish peroxide (HRP) IgG were purchased from Cell Signaling Technology (USA). Annexin V-FITC/PI Apoptosis Detection kit was acquired from Bestbio Company (China).

### Cells culture

MG63 cells were cultured with Dulbecco’s Modified Eagle Medium (DMEM) including 10% FBS, 100 mg/L streptomycin, and 100 mg/L penicillin with 5% CO_2_ at 37°C. Cells were digested with 0.25% pancreatic enzymes and passaged in a ratio of 1:3 every three days. The following experiments were set after the degrees of cell fusion attained 80%.

### Observation of morphological variations and DAPI staining

MG63 cells were incubated at a concentration of 1 × 10^5^ cells per well in six-well culture plates. Experimental groups were treated with three different concentrations (2, 5, and 8 μg/mL) of formononetin for 48 h, whereas the control group (0 μg/mL) was treated with DMEM containing 10% FBS. After treatment, morphological appearances were observed with an inverted microscope.

DAPI staining was performed to establish cell apoptosis. Treated cells were fixed with 4% paraformaldehyde solution (37°C, 15 min) and their membrane penetrability increased using 0.1% TritionX-100 solution. Subsequently, treated cells were incubated with 1 g/ml (1:1000 dilution) DAPI for 10 min and washed with Phosphate Buffer Solution (PBS). A fluorescence microscope was used to observe the nuclear morphology of PBS-washed cells.

### MTT assay

MG63 cells of logarithmic growth were used to make cell suspension and seeded at the density of 5 × 10^3^ cells/well in 96-well plates. Each well was added with 200 μL cell suspension. After incubation overnight, MG63 cells were cultured with various concentrations (0, 2, 5, 8 μg/mL) of formononetin for 48 h. Cells were then incubated with MTT at 37°C and 5% CO_2_ for 4 h. Subsequently, 150 μL DMSO was added to each well to end the reaction. Finally, plates were vortexed for 10 min, and their absorbance at 490 nm was labeled with a microplate reader.

### Cell scratch test

The effect of formononetin on adhesive and invasive abilities of MG63 cells was detected with a scratch test. First, six-well plates were marked with equal straight lines on the back with at least five lines crossing over each orifice. The cell suspension was added into plates at a concentration of about 1×10^5^ cells/well and cultured with 5% CO_2_ at 37°C overnight. Then, transverse lines perpendicular to the straight lines were drawn on the back of these plates. Next, after washing with PBS, MG63 cells were incubated with diverse concentrations (0, 2, 5, 8 μg/mL) of formononetin for 24 h. Finally, cell migration was observed and photographed with an optical microscope.

### Flow cytometric analysis

To investigate cell apoptosis, MG63 cells in the control group and experiment group were collected and rinsed severally with PBS. Cells were stained with PI and Annexin V-FITC solution for 20 min at room temperature and analyzed with flow cytometry.

### Western blotting assay

Total proteins were divided into equal amounts and separated on SDS-PAGE gels. Then, the protein in gels was transferred to PVDF membranes. The membranes were blocked with 5% BCA for 1 h. Then electroblotted PVDF membranes were incubated overnight at 4°C with 1:500 primary antibodies including bcl-2, caspase-3, p21, and p53, followed by 1:2000 diluted secondary antibodies at room temperature for 1.5 h. Blots were then developed in the dark room and scanned with Bio-Rad (USA) GS800. Finally, quantitative analysis was performed with ImageJ 1.41 software.

### Statistical analysis

All tests were repeated in three independent experiments. Data were analyzed with SPSS 16.0 and expressed as mean ± standard deviation (SD). One-way analysis of variance (ANOVA) followed by a post-hoc test was used to analyze the results. Values with *p* < 0.05 were considered statistically significant.

### Data availability statement

The original contributions presented in the study are included in the article/Supplementary Material, further inquiries can be directed to the corresponding author.

## RESULTS

### Formononetin target protein database establishment and target protein analysis

**Figure 2:**
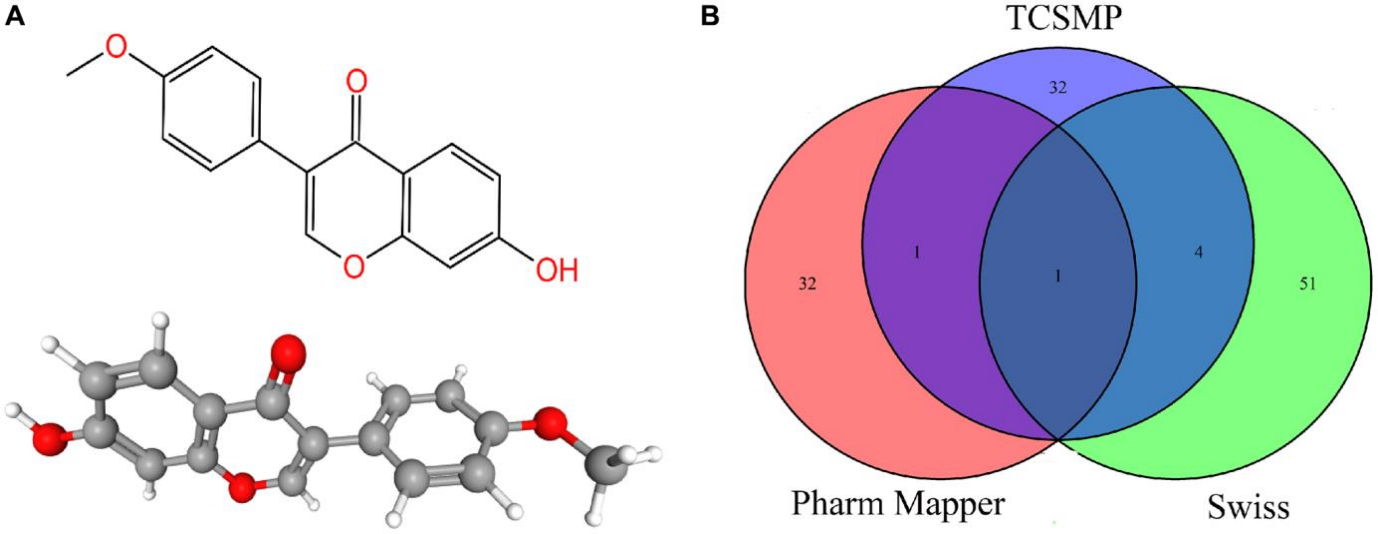
(**A**) The 2D and 3D chemical structure of formononetin. (**B**) The predicted targets of formononetin.

In total, 121 target proteins were collected from Traditional Chinese Medicine Systems Pharmacology (TCMSP), Pharm Mapper, and Swiss database. The intersecting target protein was ESR1, which revealed a relatively more significant role of ESR1 in the play (Figure 2A, 2B). Using the David website and KEGG, these targets were screened with GO enrichment analysis (Supplementary Figure 1A, 1B) and KEGG enrichment analysis (Supplementary Figure 1C, 1D). Serotonin-containing synaptic pathway (GO ID is hsa04726), one of the signal pathways of formononetin, contains 11 formononetin target genes, including *MAOA, HTR2A, HTR2C, ALOX12, ALOX15, PTGS1, MAOB, RAF1, PTGS2, PRKACA* and *SLC6A4* (Supplementary Figure 2).

### Construction of PPI network diagram and formononetin target-pathway network

**Figure 3:**
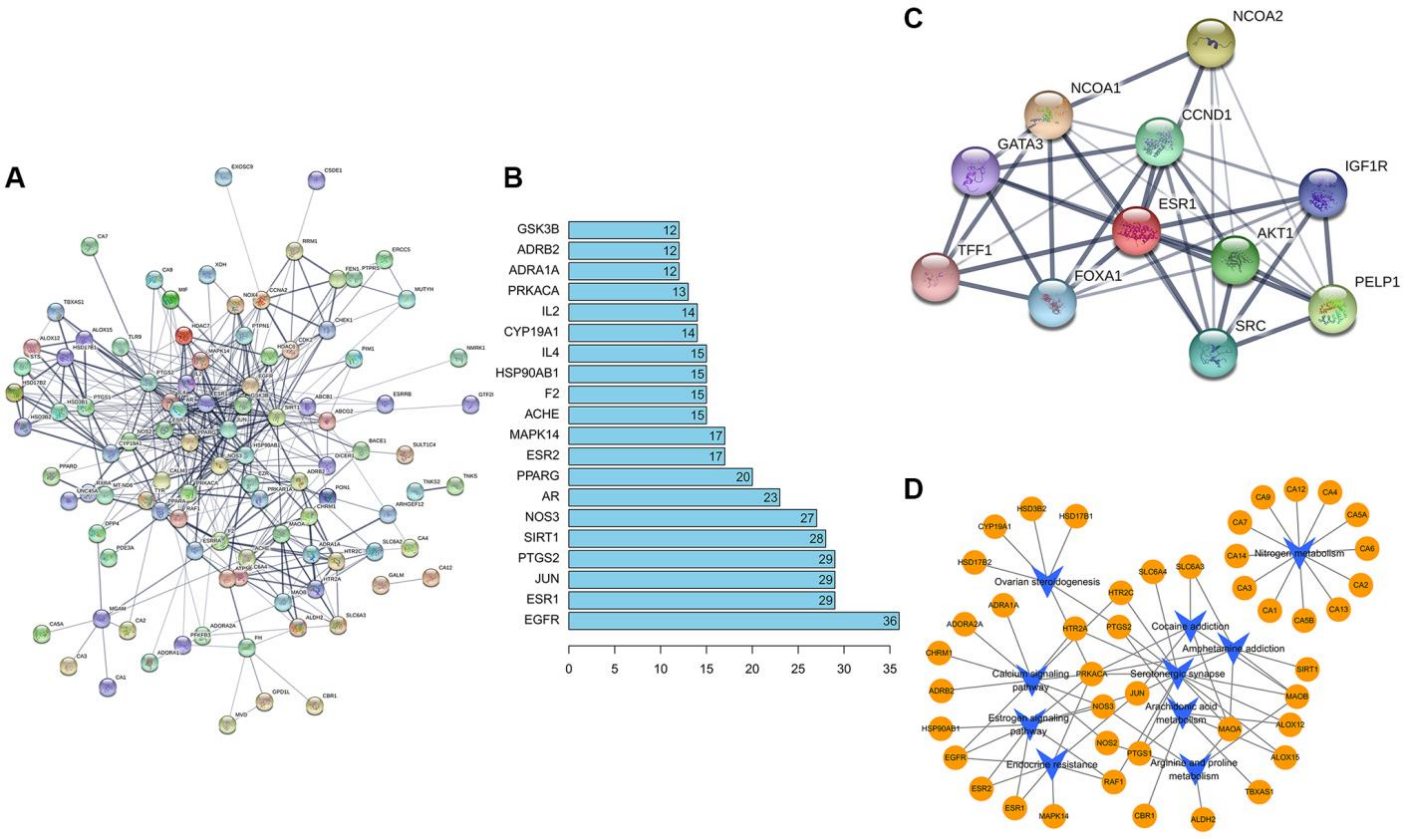
(**A**) Construction of formononetin network. (**B**) The abscissa represents the number of related pairs of genes in the network diagram. (**C**) The ESR1 protein interaction diagram. (**D**) The formononetin targets gene network.

STRING database was used to draw a PPI network diagram of the targets (Figure 3A), which presented both direct and indirect regulatory relationships between targets. PPI network diagram comprised 121 nodes and 406 edges (PPI enrichment *p*-value < 1.0e^−16^). The average node degree is 6.71. The average local clustering coefficient is 0.493. The expected number of edges is 193. The nodes represent proteins, and the connections represent interactions between proteins. The more connections there are, the greater the degree of connection. The abscissa depicted 20 gene relationship pairs in the network diagram, including EGFR, ESR1, JUN, PTGS2, SIRT1, NOS3, AR, PPARG, ESR2, MAPK14, ACHE, F2, HSP90AB1, IL4, CYP19A1, IL2, PRKACA, ADRA1A, ADRB2, and GSK3B, in descending order of the number of related pairs of genes in the network diagram (Figure 3B). Considering ESR1 is the key target of formononetin, we also did the PPI network diagram of ESR1, and Figure 3C is the ESR1 protein interaction diagram. The PPI network diagram comprised 11 nodes and 39 edges (PPI enrichment *p*-value is 4e^−0.5^). Cytoscape software was used to draw a network diagram demonstrating the pathway regulation mechanism of formononetin on related target proteins (Figure 3D). The yellow circles indicate formononetin targets, and the blue arrows indicate the KEGG pathways.

### Molecular docking verification of ESR1 protein and formononetin

**Figure 4:**
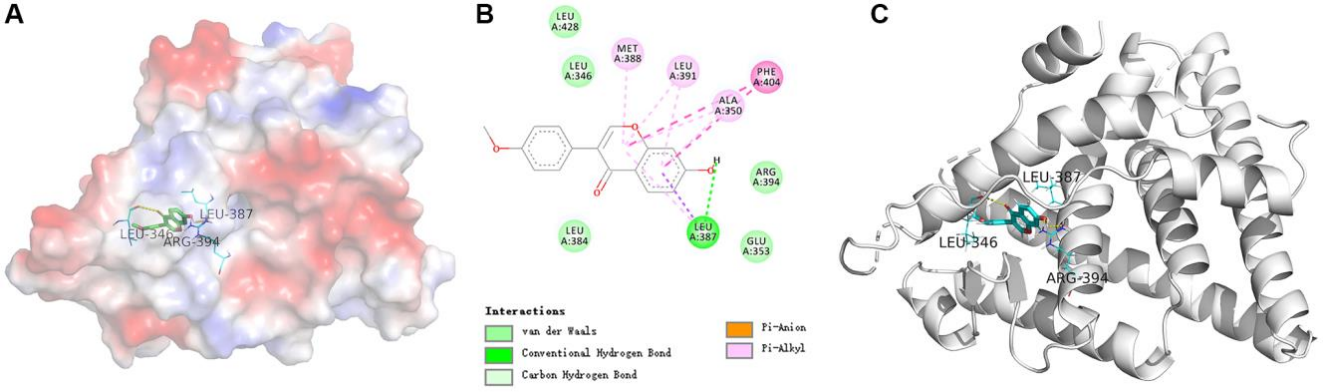
(**A**) The electrostatic potential energy diagram of ESR1 and formononetin ligand. (**B**) The 2D structural diagram showing the interaction between ESR1 and formononetin ligand. (**C**) The structural diagram illustrating the interaction between ESR1 and formononetin ligand.

In order to study the key target ESR1 deeper, the molecular docking verification of SATB2 and formononetin was performed. In a schematic diagram of docking of ESR1 protein and formononetin (Figure 4A–4C), the affinity between formononetin and the ESR1 target protein is −7.7 kcal/mol, indicating a strong interaction between ligand and receptor. The formononetin ligand forms a hydrogen bond with three residues in ESR1 protein, namely, ARG-394, LEU-346, and LEU-387.

### Screening and validation of OS target genes

**Figure 5:**
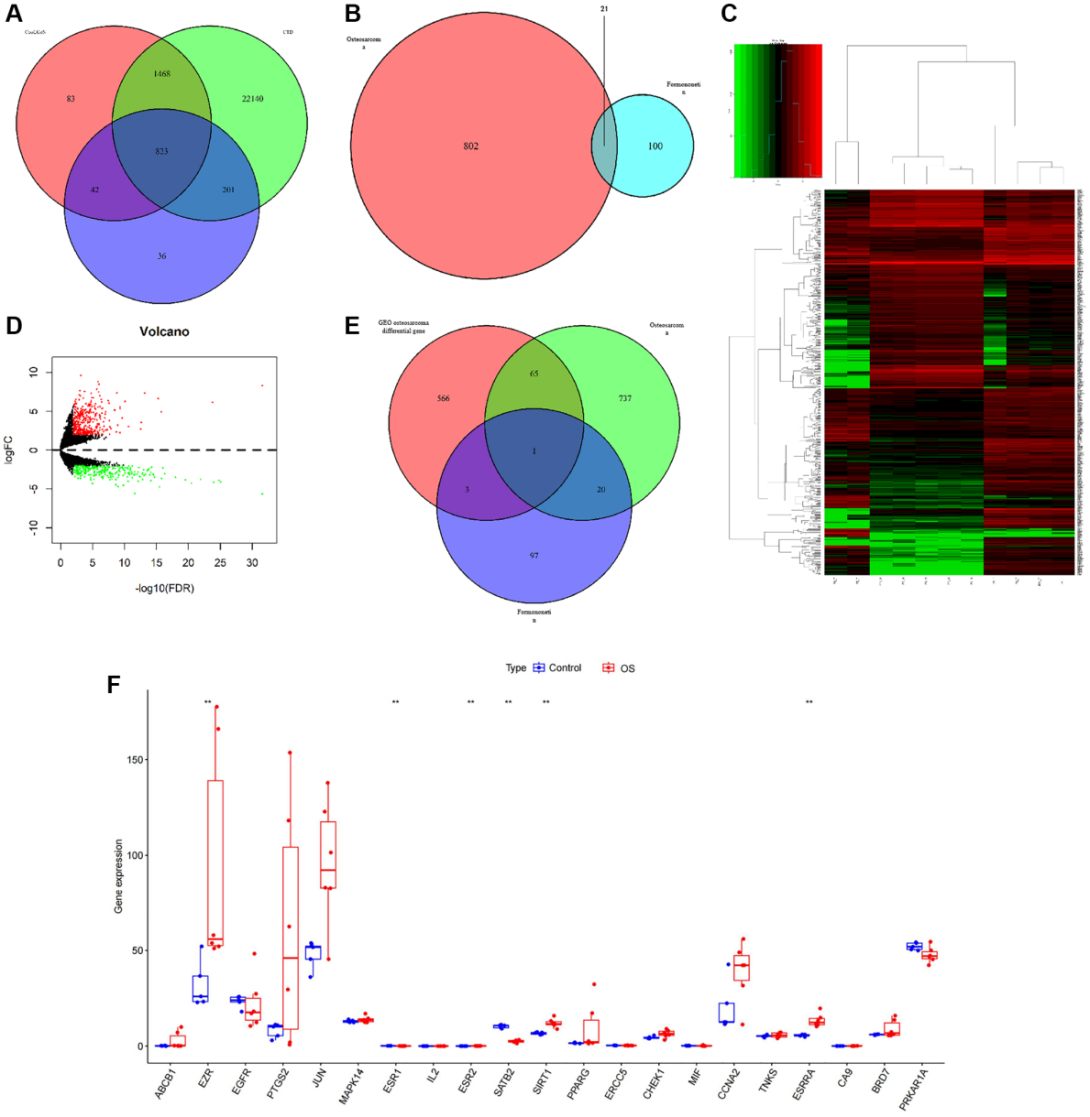
(**A**) A Venn diagram of the predicted targets of OS. (**B**) A Venn diagram of OS and formononetin targets. (**C**, **D**) A calorimetric map and differential gene volcanic map for the OS group and normal group based on GSE126209 chip in the GEO database. (**E**) A Venn diagram showing the prediction targets for the OS in the GEO database. (**F**) A boxplot extracting the expression levels in the GEO database.

We explored a total of 823 target proteins from CTD, CooLGeN, and DisGeNET databases (Figure 5A). In total, 21 common prediction targets were searched from combined formononetin targets collected from TCMSP, Pharm Mapper, and Swiss database and intersection targets of OS from CTD, CooLGeN, and DisGeNET database (Figure 5B). GEO dataset was used to validate the target protein. In total, 635 differential genes were searched from Chip GSE126209. Of these, 367 were up-regulated whereas 268 were down-regulated (Figure 5C, 5D). SATB2 protein target was identified from predicted formononetin intersection targets, OS targets, and GEO differential genes (Figure 5E). A boxplot extracted the expression levels in the GEO database (Figure 5F). Comprehensively, we considered that the STAB2 might serve as the key target in treating osteosarcoma.

### GO and KEGG analysis of formononetin anti-OS targets

**Figure 6:**
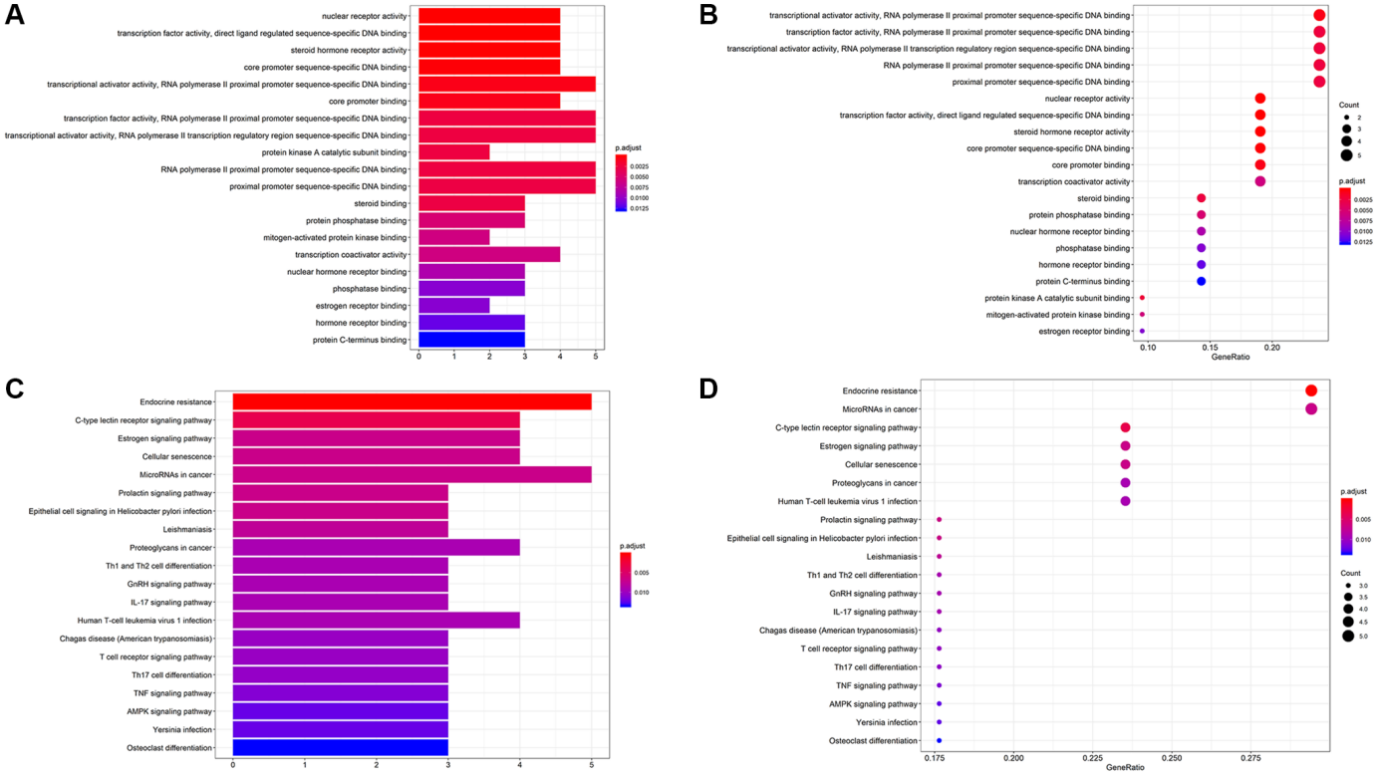
(**A**, **B**) The bar chart and bubble chart showing top 20 GO ID. (**C**, **D**) The bar chart and bubble chart showing the top 20 KEGG pathways of potential target genes of Form in OS.

We performed GO enrichment analysis (Figure 6A, 6B) and KEGG enrichment analysis (Figure 6C, 6D) on 21 prediction targets common to formononetin anti-OS through the David website and KEGG. Only the top 20 GO IDs are shown. These targets were involved in Endocrine resistance pathways, MicroRNAs in the cancer pathway, C-type lectin receptor signaling pathway, Estrogen signaling pathway, and Cellular senescence pathway.

### Construction of PPI network diagram and formononetin target-pathway network

**Figure 7:**
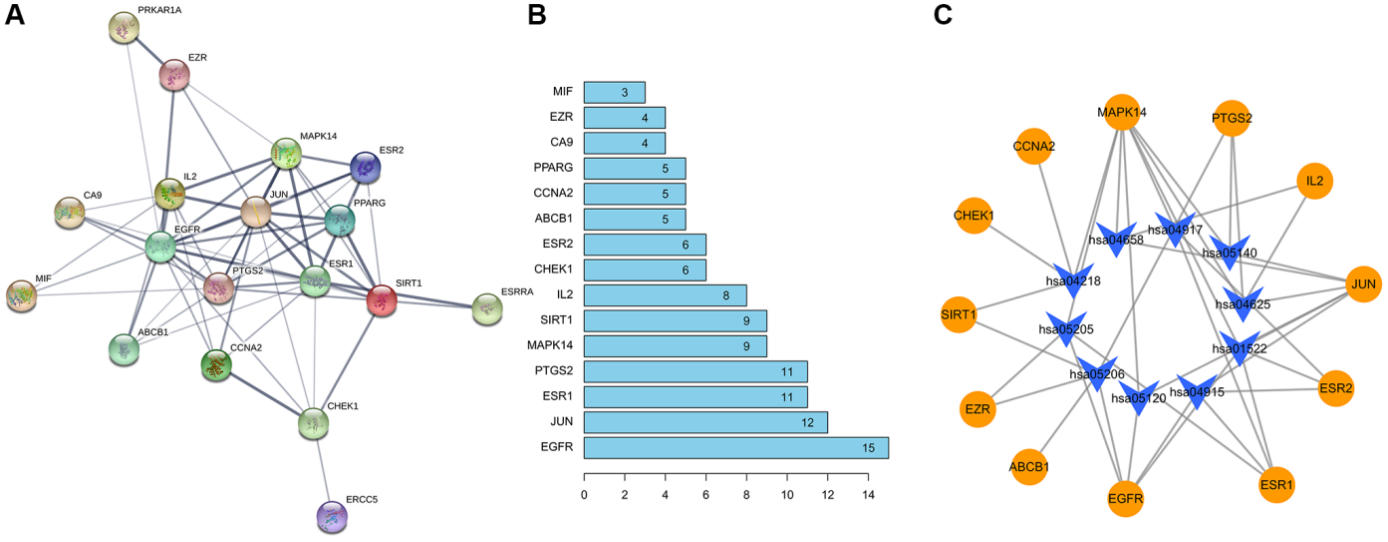
Construction of PPI network diagram (**A**) The protein interaction diagram. (**B**) The abscissa represents the number of related pairs of genes in the network diagram. (**C**) The formononetin-OS target gene network.

STRING database was used to draw a PPI network diagram of the targets (Figure 7A) demonstrating direct and indirect regulatory relationships between targets. The Number of nodes is 21. The Number of edges is 59. The average node degree is 5.62. Average local clustering coefficient is 0.648. The expected number of edges is 19. PPI enrichment *p*-value is 3.86e^−13^. The nodes represent proteins, and the connections represent interactions between proteins. The more connections there are, the greater the degree of connection. In core gene analysis results (Figure 7B), the abscissa depicted 15 gene relationship pairs in the network diagram, namely EGFR, JUN, ESR1, PTGS2, MAPK14, SIRT1, IL2, CHEK1, ESR2, ABCB1, CCNA2, PPARG, CA9, EZR, MIF in descending order of the number of related pairs of genes in the network diagram. Cytoscape software was used to draw a pharmacology network diagram demonstrating the pathway regulation mechanism of formononetin-OS-related protein targets based on KEGG pathway analysis (Figure 7C). The yellow circles indicate formononetin-OS prediction targets, and the blue arrows indicate the KEGG pathways.

### Molecular docking verification of SATB2 and formononetin

**Figure 8:**
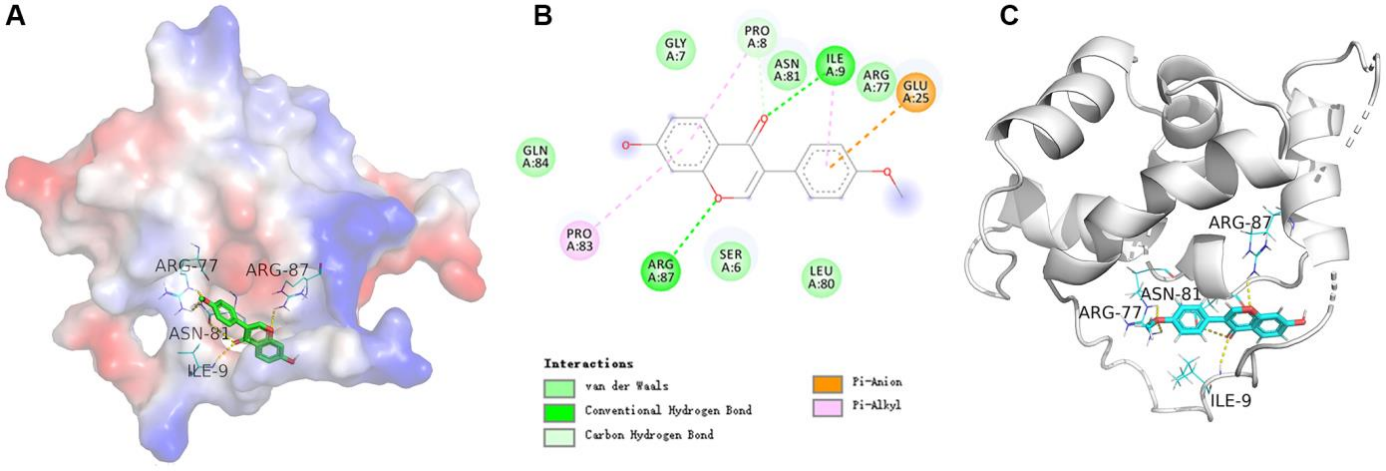
(**A**) The electrostatic potential energy diagram of SATB2 and formononetin ligand. (**B**) The structural diagram showing the interaction between SATB2 and formononetin ligand. (**C**) The 2D structural diagram illustrating the interaction between ESR1 and formononetin ligand.

In order to study the key target SATB2 further, the molecular docking verification of SATB2 and formononetin was performed. In a schematic diagram of docking of SATB2 protein and formononetin (Figure 8), the affinity between formononetin and SATB2 target protein was −7 kcal/mol, indicating a strong interaction between SATB2 and formononetin. The formononetin ligand formed five hydrogen bonds with four residues in SATB2 protein, including ARG-87, ASN-81, ARG-77, and ILE-9.

### Effects of formononetin on morphology and DAPI staining of MG63 cells

**Figure 9:**
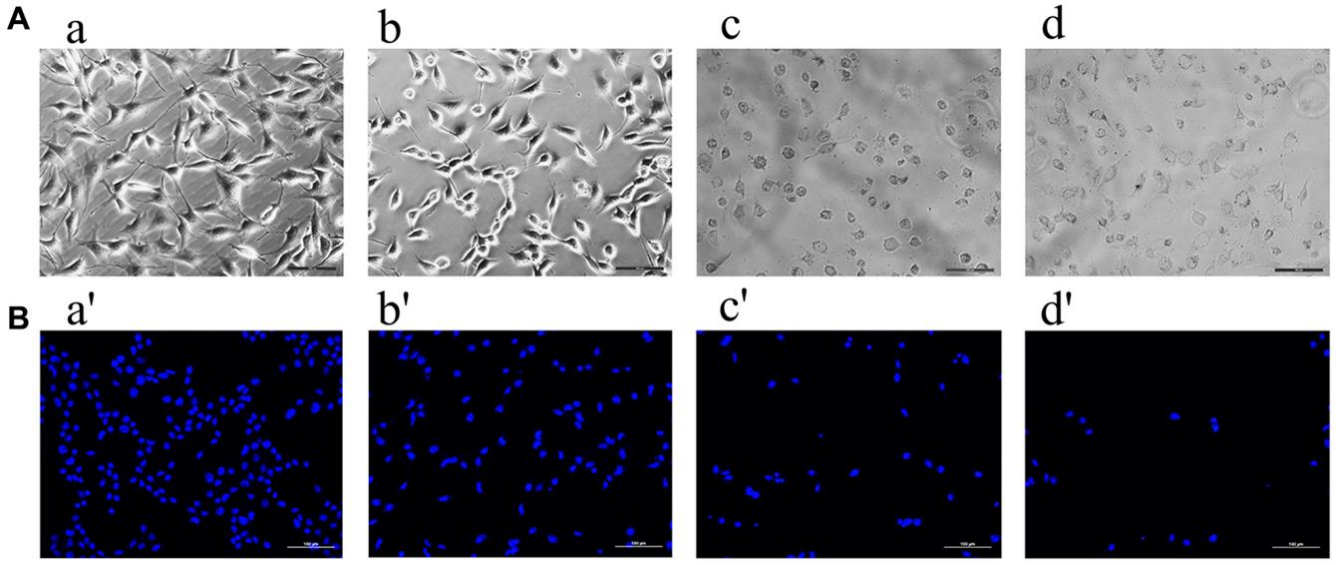
The ((**A**) **a**–**d**) showed the results observed under the inverted microscope after being treated by different concentrations of formononetin for 48 h. Meanwhile, ((**B**) **a′**–**d′**) showed the results treated with DAPI for 48 h to assess cell apoptosis. (**a**, **a′**) The MG63 cells were treated with 0μg/mL Form for 48 h (the control group). (**b**, **b′**) The MG63 cells were treated with 5 μg/mL Form for 48 h. (**c**, **c′**) The MG63 cells were treated with 8 μg/mL Form for 48 h. (**d**, **d′**) The MG63 cells were treated with 10 μg/mL Form for 48 h.

When observed under an optical microscope, MG63 cells in the control group were intact, spindle-shaped, adhered to the wall, and had unclear cell boundaries (Figure 9A (a)). Treatment with 2 μg/mL, 5 μg/mL or 8 μg/mL formononetin for 48 h induced cell apoptosis. The cells appeared shrunk and spherical (Figure 9A (b–d)). Compared with the control group, apoptosis in experimental groups increased in a concentration- dependent manner. Cells were treated with DAPI for 48 h to assess cell apoptosis. Likewise, an intact nucleus and no evidence of apoptosis was observed in no-formononetin-treated control group. However, in the experimental groups treated with 2 μg/mL, 5 μg/mL, and 8 μg/mL formononetin for 48 h, apoptosis was observed (Figure 9B (a′–d′)).

### Effects of formononetin on proliferation of MG63 cells

**Figure 10:**
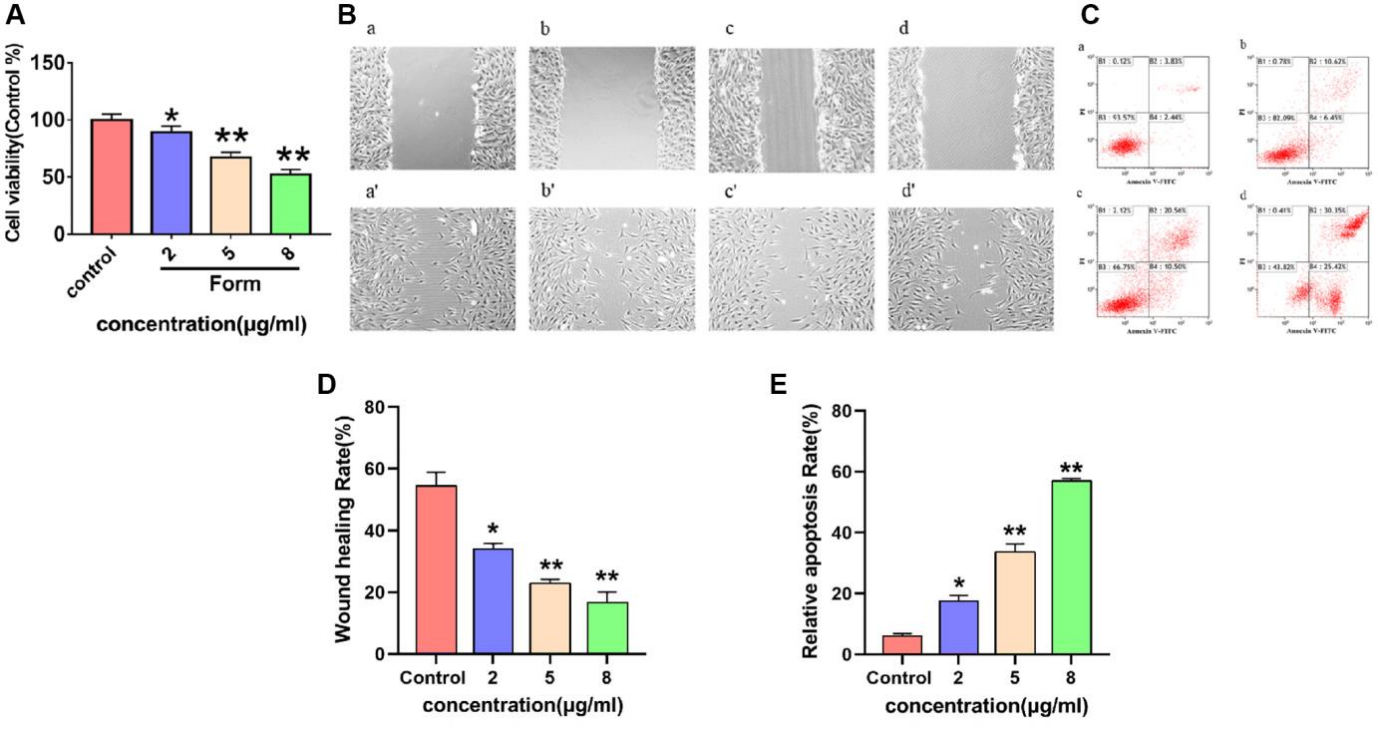
(**A**) The inhibitory effect of different concentrations of formononetin (0, 2, 5, 8 μg/mL) on the proliferation of MG63 cells. Data are presented as the means ± SD of three independent experiments. ^*^p < 0.05, ^**^p < 0.01 vs. control group. (**B**) Images of the wounds of cell layer at 0 h (**a**–**d**) and after 48 h (**a**′–**d**′), showing the width of scratches with the effect of different concentrations of formononetin (0, 2, 5, 8 μg/mL) on the migratory ability of MG63 cells. (**C**) Representative results of Annexin-V/PI staining for formononetin. (**a**–**d**) Cells were treated with 0, 2, 5, 8 μg/mL formononetin for 48 h. (**D**) Wound healing assay of formononetin treated MG63 cells. (**E**) The apoptosis rate of MG63 cells after formononetin treatment.

To verify whether formononetin could inhibit cell proliferation, MG63 cells were cultured with diverse concentrations of formononetin (0, 2, 5, and 8 μg/mL) for 48 h. Cell proliferation was assessed using an MTT assay. The results showed that cell survival rate declined with increasing formononetin concentration (Figure 10A), demonstrating that formononetin has an inhibitory effect on MG63 proliferation.

### Effect of formononetin on migration of MG63 cells

The effect of different concentrations of formononetin (0, 2, 5, and 8 μg/mL) on the migration ability of MG63 cells was analyzed using cell scratch test. As shown in Figure 10B (a, a′–d, d′) and 10D, the cell migration was significantly accelerated in the control group compared with the experimental groups after 48 h.

### Effect of formononetin induced apoptosis in MG63 cells

Flow cytometry analysis was performed to confirm apoptotic cell damage. In the control group, few apoptotic cells were detected (Figure 10C (a)). However, cells treated with 2 μg/mL formononetin exhibited remarkable apoptosis of up to 17.07% (Figure 10C (b)). In addition, treatment with 5 μg/mL and 8 μg/mL formononetin for 48 h increased apoptotic rate to 31.06% and 55.77%, respectively (Figure 10C (c, d) and 10E).

### Formononetin stimulated the mitochondrial pathway and inhibited the expression of SATB2

**Figure 11:**
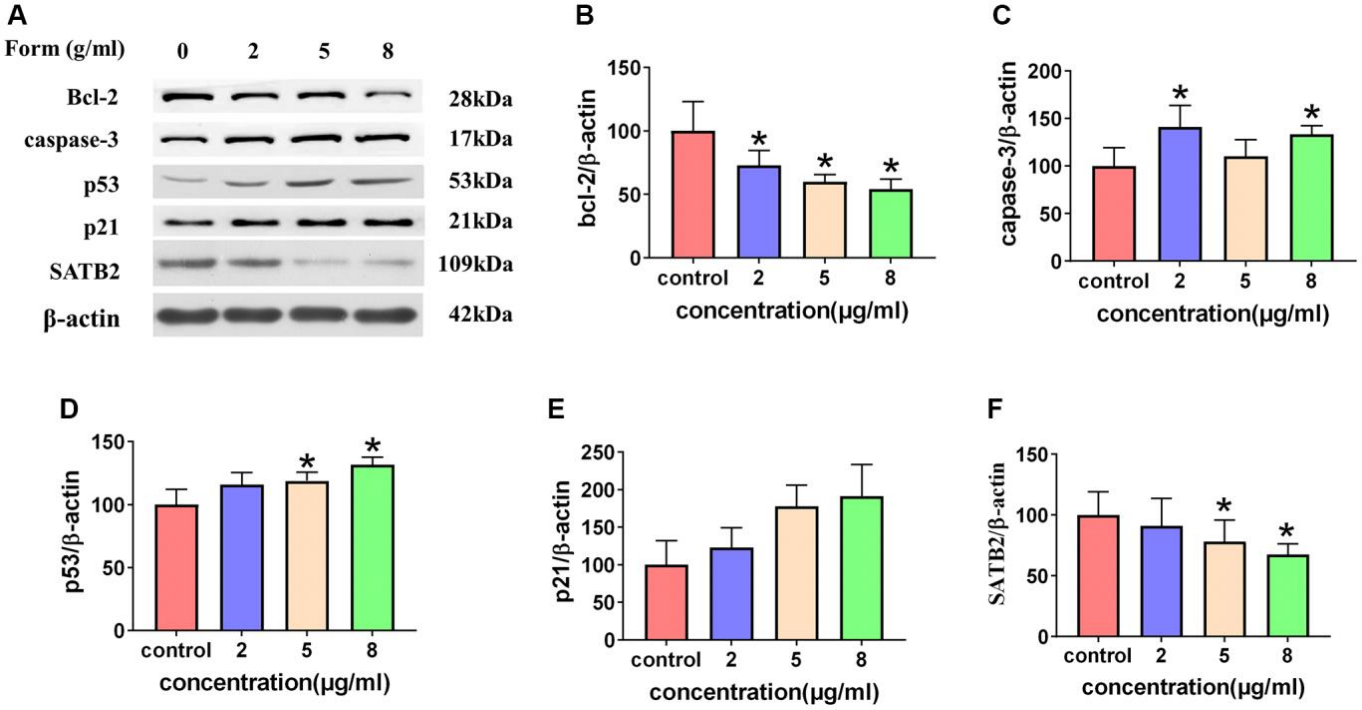
(**A**) Western blotting results showing expression of cleaved caspase-3, p21, and p53. Formononetin inhibited bcl-2 expression (**B**) and induced the expression of cleaved caspase-3 (**C**) through the p21/p53 (**D**, **E**) pathway and inhibited the expression of SATB2 (**F**). Data are presented are the means ± SD of three independent experiments. ^*^p < 0.01 vs. control group.

Cellular levels of bcl-2, caspase-3, p53, p21, and SATB2 protein in different concentrations of formononetin (0, 2, 5, and 8 μg/mL) were demonstrated in MG63 cells using a western blotting assay (Figure 11). Analysis of cell cultures incubated with various concentrations of formononetin for 48 h showed a decrease in gray ratios of bcl-2 with increasing concentrations of formononetin. Further, experimental groups had significantly lower gray ratios of bcl-2 than the control group. Expression levels of caspase-3, p53, and p21 in the experiment groups were higher than in the control group and increased concomitantly with formononetin concentrations. The cellular level of SATB2 protein in different concentrations of formononetin (0, 2, 5, 8 μg/mL) after 48 h treatment was suppressed in MG63 cells as shown through western blotting assay. The gray ratio of SATB2/β-actin of the experimental groups was significantly lower than that of the control group and decreased gradually as the drug dose was increased.

## DISCUSSION

Osteosarcoma (OS), the most prevalent malignant disease of primary bone, occurs predominantly in children and young adults with high morbidity and easy metastasis. Evidence from previous studies indicates that chemotherapy, radio therapy, and surgery are the main therapeutic strategies for OS [27]. Experimental studies have demonstrated that multiple mechanisms contribute to the progression of OS [28, 29]. Therefore, it is of great value to find tumor drugs with high efficacy but minimal side effects.

In this study, network pharmacology was employed to analyze formononetin as a potential anti-OS drug. Our database search yielded 121 prediction targets and ESR1 as the core target, which had a strong interaction effect with formononetin. ESR1 is a ligand-dependent transcription factor estrogen receptor. When ESRI binds with its ligands, it stimulates related signal transduction pathways involved in the growth and development, and apoptosis of tumor cells [30, 31]. The classical estrogen receptor signal transduction pathway is that estrogen directly binds to nuclear estrogen receptor ESR1 and activates the transcription of target genes [32]. Studies have shown that the abnormal estrogen signaling pathway affects the normal expression of related genes, resulting in abnormal proliferation and apoptosis of tumor cells [33]. KEGG analysis indicated that formononetin affected many pathways, including the nitrogen metabolism pathway, serotonergic synapse pathway, and ovarian steroidogenesis. Furthermore, we explored possible targets of formononetin anti-OS. Out of 823 prediction targets we collected, 21 targets were common to formononetin and OS. KEGG analysis of these 21 targets showed that bioactive ingredients of formononetin were involved in endocrine resistance pathways, microRNAs in cancer pathway, C-type lectin receptor signaling pathway, estrogen signaling pathway, and cellular senescence pathway. Recent research suggests that endocrine resistance pathways play an important role in the development and treatment of cancer [34]. C-type lectin receptor deficiency has also been postulated to inhibit thrombus formation in tumor vessels, improving oxygen and nutrient supply to tumors and thereby indirectly promoting tumor proliferation [35]. We found four targets including EGFR, JUN, ESR1 and PTGS2 in the constructed PPI network. In agreement with our analysis results, recent research results have shown that EGFR plays a significant role in the development of OS. EGFR phosphorylation is involved in the migration and invasion of OS mediated by Circular RNA cir-ITCH [36]. What’s more, it’s also researched that tumor-suppressive activity in OS is correlative with the downregulation of EGFR [37, 38]. Through validation of target protein in the GEO dataset, we obtained SATB2 protein target exhibiting strong interaction with formononetin. SATB2 is a DNA binding protein that specifically binds to the nuclear matrix attachment region regulates transcription of higher chromatin domain and is highly sensitive and specific to osteoblastic tumors, non-small cell lung cancer, breast cancer, and colorectal cancer [39–42]. Previous osteosarcoma studies have shown that targeting SATB2-mediated EMT could inhibit OS cell growth and metastasis [40]. In our study, the western blotting assay showed that different concentrations of formononetin (0, 2, 5, 8 μg/mL) suppressed cellular level of SATB2 protein in MG63 cells after 48 h treatment, partially consistent with the predicted mechanism of network pharmacology.

Currently, studies have found that the occurrence of both phytoestrogens and osteosarcoma cells influences MG63 cell proliferation, differentiation, apoptosis, invasion, and migration [16]. Organic cation transporter 2 (Oct 2) was downregulated and drug-fast associated proteins were elevated after formononetin treatment. Formononetin also regulated cyclins and pro-apoptotic proteins involved in the cell cycle [43]. In recent studies, formononetin has been shown to have a certain inhibitory effect on OS. Liu and his colleagues discovered that formononetin promoted apoptosis of U2OS cells [9]. In this study, we assessed the effects of formononetin on the proliferation and apoptosis of MG63 cells. Our results indicated that formononetin accelerates apoptosis of MG63 cells and has some inhibitory effect on OS. Therefore, formononetin is a potential novel therapeutic candidate for the treatment of malignant OS tumors.

Cell proliferation is an important physiological function of living cells and a key feature of living organisms. Cells usually reproduce by differentiation to replenish cells lost through aging or cell death [14]. Normal cells exhibit a mechanism for controlling cell differentiation and proliferation that is lacking in tumor cells. More importantly, malignant proliferation occurs only in tumor cells. Therefore, inhibiting the malignant proliferation and migration of tumor cells is a promising therapeutic approach to controlling the occurrence and development of malignant tumors [28]. In our study, we used the MTT assay to detect the viability of human MG63 cells treated with various concentrations of formononetin. The results suggested that formononetin inhibits the ability of MG63 cells to proliferate. Interestingly, when the concentration of formononetin was increased, its inhibitory effect on MG63 cells also increased gradually, demonstrating a significant dose-dependent relationship. Meanwhile, we found that the maximum concentration that could inhibit MG63 proliferation was 8 μg/mL. Therefore, formononetin could be used to inhibit MG63 cell proliferation *in vitro* within a certain concentration range.

Apoptosis is an active process of cell death controlled by genes to better adapt the organism to its environment. The initiation and development of tumors are mainly due to apoptotic dysfunction. Studies have shown that most anti-tumor drugs inhibit tumor growth by inducing apoptosis in tumor cells [44]. For instance, Liu et al. found that formononetin significantly induced cytotoxicity in human osteosarcoma cells and had the excellent therapeutic potential for OS. In the present study, it was further demonstrated that formononetin could effectively induce apoptosis of MG63 cells in a time- and dose-dependent manner.

**Figure 12:**
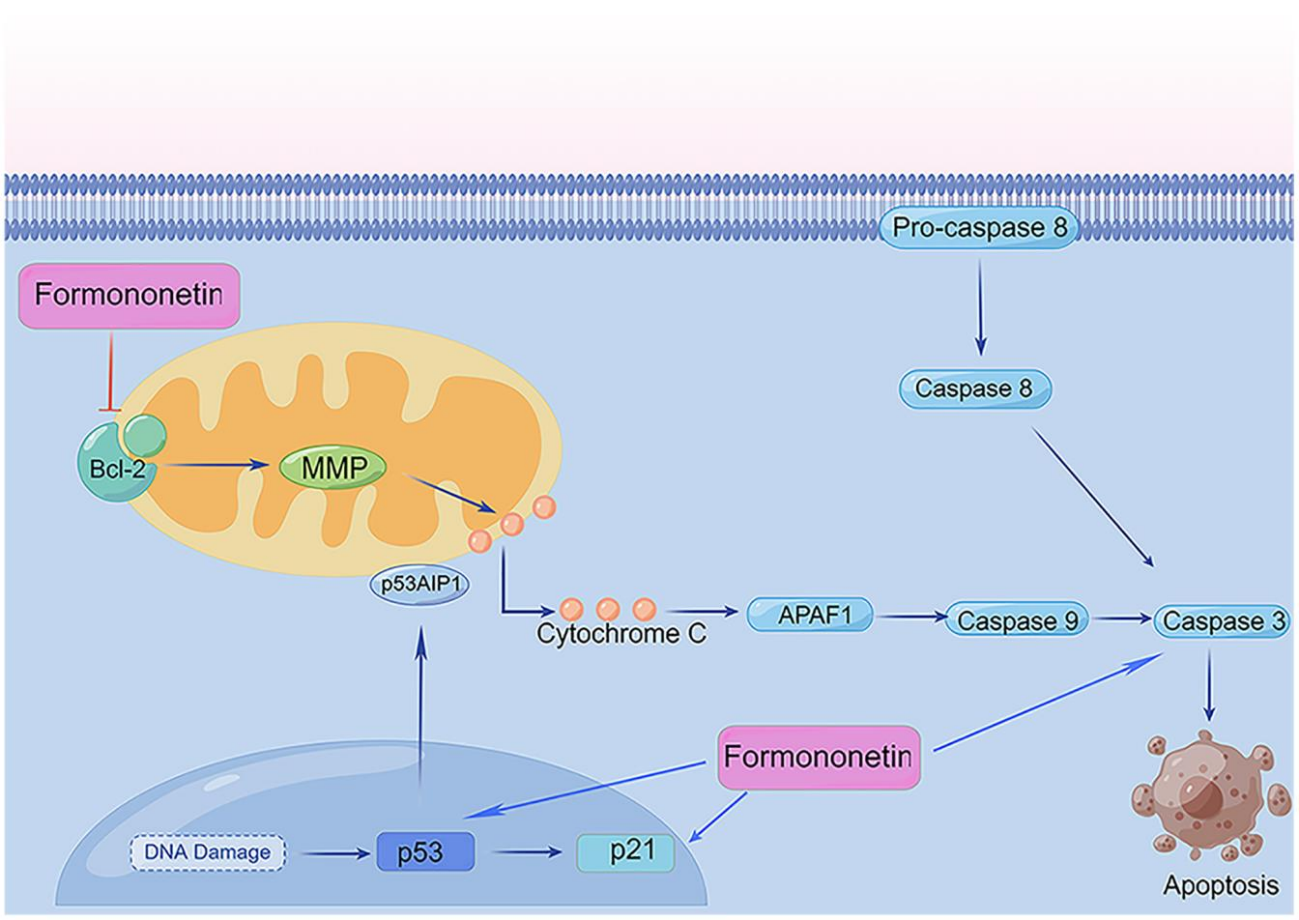
**Formononetin regulates apoptosis-related mitochondrial pathways in MG63 cells.** ⊥, inhibition/downregulation; ↑, upregulation/activation. By Figdraw (http://www.figdraw.com/).

Multiple signal transduction pathways are involved in apoptosis, including the mitochondrial pathway that has been implicated apoptotic transduction in the organism. Mitochondria are the control center of vital cellular activities and regulate apoptosis of cells [45]. Bcl-2 is the upstream signaling molecule of caspase-3 regulating cell apoptosis. Within the caspase-protease family, caspase-3 is the promoter and inducer of apoptosis [46]. Once the apoptotic program has been initiated, caspase-3 is activated and then promotes apoptosis. Besides, p53 is a type of tumor suppressor protein strongly associated with the occurrence of tumors. P53 regulates the cell cycle and prevents cell carcinogenesis [47]. In contrast, p21 is a cyclin-dependent kinase inhibitor occurring downstream of p53. Research has shown that p21 is indirectly involved in apoptosis through the p53-dependent pathway [48]. In the present study, the mechanism of formononetin promoting apoptosis was studied by western blotting analysis. Our results revealed that expression of caspase-3, p21, and p53 were markedly up-regulated by formononetin. Meanwhile, bcl-2 expression indicated a formononetin concentrations-dependent decrease trend significantly. The findings indicated that formononetin could activate the bcl-2 signaling pathway. Therefore, our results support the effect of formononetin on the mitochondrial pathway in MG63 cells (Figure 12).

In conclusion, our experiment used network pharmacology to explore the pharmacological mechanism of formononetin and the mechanism of formononetin anti-OS, providing a new research direction for the pathogenesis of osteosarcoma and a potential therapeutic target for OS. Furthermore, we verified the anti-tumor activity of formononetin through experiments showing that it inhibits the proliferation of MG63 cells in a concentration-dependent manner and induces the apoptosis of MG63 cells. Furthermore, our results confirmed the typical morphological changes in apoptotic cells and the variation of apoptosis-related protein. These findings suggest that apoptosis may be related to the down-regulation of bcl-2 and up-regulation of caspase-3, p21, and p53. In summary, we identified that formononetin could be a potential anti-tumor approach in the therapy of osteosarcoma. Further studies are needed to provide more insights into the pharmacological mechanism of formononetin.

## Supplementary Materials

Supplementary Figures
