# Supplementary material for: Anti-cancer targets and molecular mechanisms of formononetin in treating osteosarcoma based on network pharmacology
Source: Aging (Albany NY). 2023 Oct 20;15(20):11489–507. doi: 10.18632/aging.205139 (PMC10637808; doi:10.18632/aging.205139)
Supplement: Supplementary Figures [file aging-15-205139-s001.pdf]

SUPPLEMENTARY FIGURES

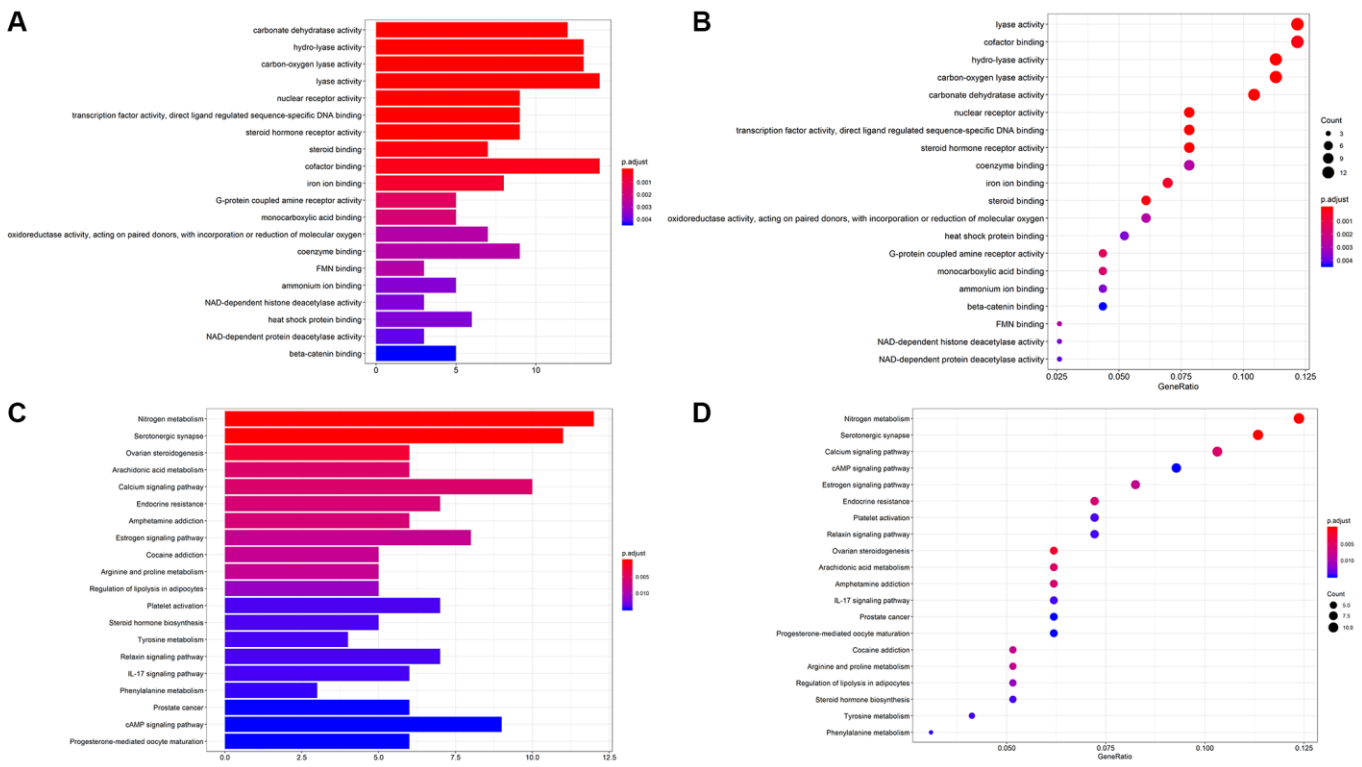

Supplementary Figure 1. (**A**, **B**) The bar chart and bubble chart showing top 20 GO ID. (**C**, **D**) The bar chart and bubble chart showing the top 20 KEGG pathways of potential target genes of Form in OS.

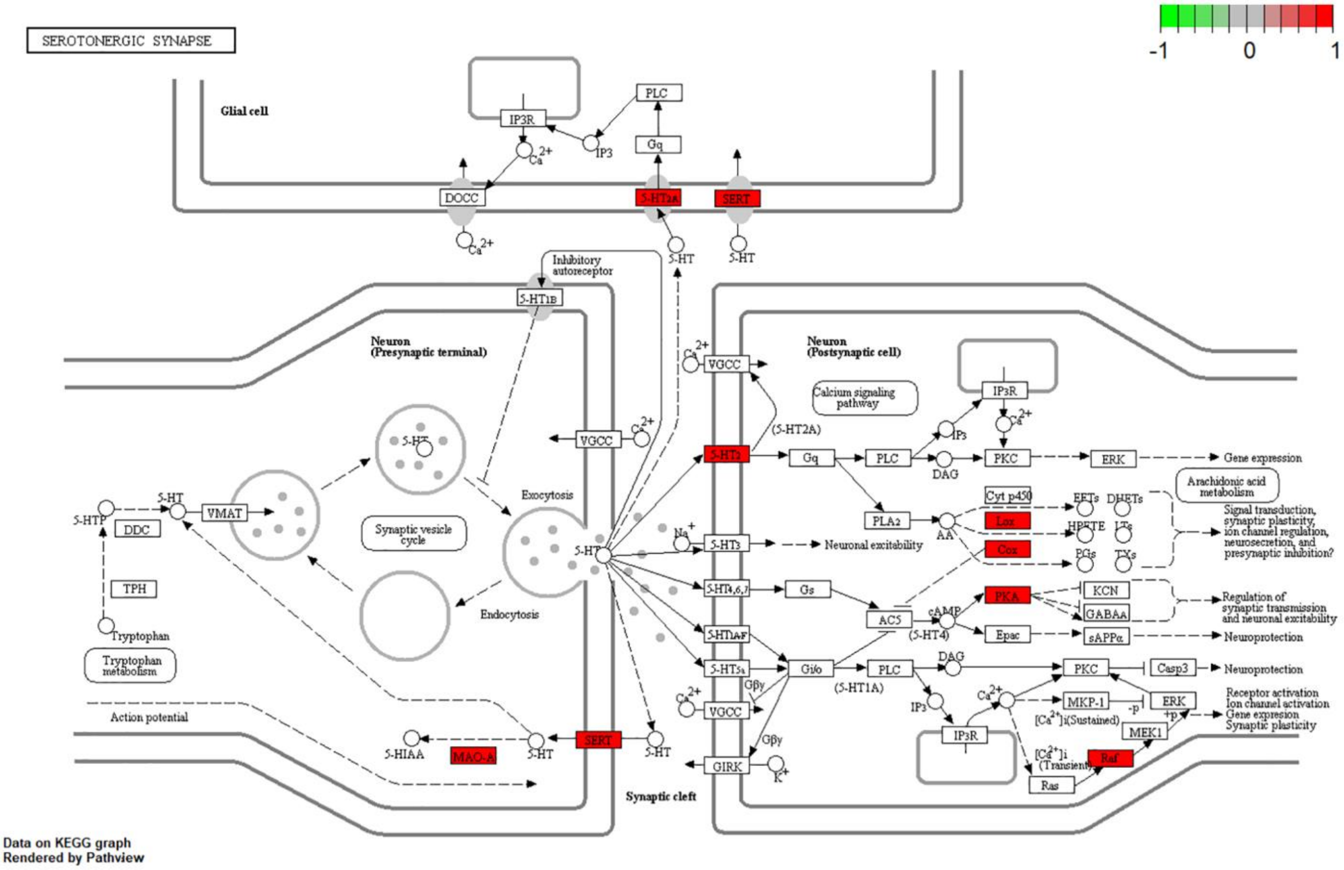

Supplementary Figure 2. Serotonin containing synaptic pathway in the KEGG pathway map. Red represents the predicted target genes of Form in the pathway.
